# Results at 3-year follow-up of totally extraperitoneal (TEP) hernia surgery with long-term resorbable mesh

**DOI:** 10.1007/s10029-019-02116-2

**Published:** 2020-01-10

**Authors:** F. Ruiz-Jasbon, K. Ticehurst, J. Ahonen, J. Norrby, P. Falk, M.-L. Ivarsson

**Affiliations:** 1 Department of Surgery, Halland’s Hospital, Kungsbacka, Sweden; 2 Department of Surgery, Institute of Clinical Science, Sahlgrenska Academy at University of Gothenburg, Göteborg, Sweden

**Keywords:** Inguinal hernia, Chronic pain, Resorbable mesh, Recurrence, TEP, Implant

## Abstract

**Introduction:**

Synthetic non-resorbable mesh is almost standard in hernia surgery. However, several studies have showed negative effects of permanent implants such as chronic inflammation and complications involving different organs bordering the mesh. Such complications can raise the risk of chronic post-operative pain (CPP). Recently promising results regarding CPP have been published in patients with Lateral Inguinal Hernia (LIH) using a slowly resorbable mesh in Lichtenstein technique. For this reason the aim of the present study was to find the effect of a slowly resorbable implant on the long-term rate of hernia recurrence and chronic post-operative pain in patients with LIH repaired with TEP procedure.

**Methods:**

Prospective pilot study of TEP repair using TIGR^®^ Matrix Surgical Mesh in 35 primary LIH. At 3-year follow-up the Visual Analogue Scale (VAS) and the Inguinal Pain Questionnaire were employed to assess pain. Recurrence was determined by ultrasound and clinical examination.

**Results:**

All patients completed the pain questionnaires but one patient did not attend the planned clinical examination for the 3-year follow-up. No patients had CPP, as defined in the World Guidelines for Groin Hernia Management. Almost all patients had lower VAS score in any activity 3 years following surgery in comparison to the preoperative period. Three patients (8.8%) suffered symptomatic recurrence during the 3-year follow-up.

**Conclusion:**

TEP repair in patients with LIH using a synthetic long-term resorbable mesh was found to be encouraging respecting chronic post-operative pain at 3-year follow-up but at the cost of an increased risk of recurrence.

## Introduction

The use of permanent synthetic implants in hernia surgery has led to a powerful reduction in the recurrence rates [1–3]. However, chronic post-operative pain (CPP) and discomfort after hernia repair with synthetic meshes continue to be serious complications for numerous patients [4, 5]. Furthermore, complications in hernia surgery with permanent implants have become a sensible issue in medico legal allegation in some countries [6, 7]. Other former surgical methods without prosthesis, like herniorrhaphy, have similar risk of CPP to hernioplasty with mesh but with higher recurrence rates in adult patients [1, 8, 9]. Thus there is a need to test new operation techniques or meshes in order to reduce the incidence of post-operative pain, while maintaining a low level of hernia recurrences.

Biological and synthetic degradable meshes have been tested in the last few years in order to reduce the risk of CPP. Some biological meshes have shown relatively good results on recurrence and pain in small studies [10, 11]. The higher cost of those biological meshes is perhaps the reason why larger randomized studies with them are not available and why biological meshes have not been used on a large scale [12]. Resorbable meshes with short resorption time have also been tested in the last decennium. A pilot study using a short term degradable implant in the Lichtenstein approach found a high recurrence risk in both Lateral Inguinal Hernia (LIH) and Medial Inguinal Hernia (MIH) 3 years after surgery [13].

Synthetic implants with longer resorption time used in open hernia surgery have been proven to be an alternative way to prevent CPP. A pilot study using such an implant, the TIGR Matrix Surgical Mesh, in the Lichtenstein technique showed good results on chronic pain and recurrences in patients with LIH at a 3-year follow-up but a high rate of recurrences in patients with MIH [14]. Since various studies indicate that the Totally Extra Peritoneal (TEP) technique has less risk of CPP than the Lichtenstein hernia repair [15, 16], our research group has recently tested the same long-term resorbable mesh in patients with LIH using the TEP technique. The trial showed promising results on chronic post-operative pain and low recurrences at 1-year follow-up [17]. The aim of the present pilot study is to perform a 3-year follow-up of the same cohort of patients in order to establish the long-term risk of recurrences and CPP in patients operated with TEP using a slowly degradable mesh.

## Methods

### Patients

**Fig. 1:**
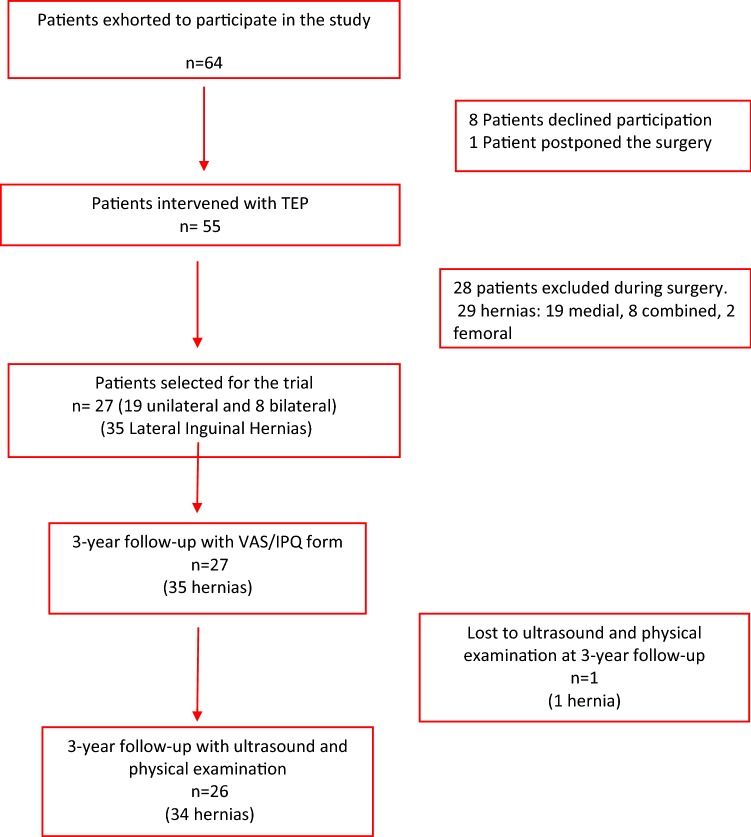
Flow chart of recruitment and follow-up of the patients

During June 2014 and February 2015 patients consulting for groin hernia and suitable for TEP operation at the department of surgery at Halland’s Hospital/Kungsbacka, Sweden, were asked to participate in this prospective study. Twenty-seven male adult patients with thirty-five primary unilateral or bilateral inguinal hernias were included in the trial. Exclusion criteria were: BMI > 35, irreducible hernias, pre-existing inguinal pain nondependent of the hernia and unwilling or unable to give informed consent. More information about enrollment and the recruitment of the patients in the study is shown in Fig. 1.

### Statement of Human and animal rights

The study was approved by the Regional Ethical Review Board in Lund, Sweden. Protocol 2014/2. All the patients enrolled in the trial provided informed consent. The study does not include any animal trial.

### Surgery

Three surgeons, with extensive experience on the TEP technique, performed the operations under general anesthesia. During the surgery patients with Femoral, Medial Inguinal or Combined Inguinal Hernias, and patients with any medial bulging were excluded from the trial and received a perdurable mesh. Patients with LIH were implanted with a 10 × 15 cm TIGR^®^ Matrix Surgical Mesh. The description of the hernia was done according to the Classification of the European Hernia Society (EHS) and the Swedish Hernia Register (SBR). An extended description of the operation technique has been previously published [17].

### Assessment at the 3-year follow-up

Pain was reported before and after surgery using two pain questionnaires validated for hernias [18, 19], the Visual Analogue Scale (VAS) 0 to 10 at different activities and the Inguinal Pain Questionnaire (IPQ). Patients with bilateral hernias were counted twice because they answered a pain questionnaire for each side. The study used the “World Guidelines for Groin Hernia Management” definition of chronic post-operative pain [20]: Moderate or higher pain intensity, persisting 3 months after surgery and affecting daily activities. This means in the present study: pain estimated as VAS ≥ 3 that cannot be ignored and that affects any daily activity.

Recurrences were assessed by both physical examination by a surgeon and ultrasound performed by a radiologist experienced in hernia diagnostics.

### Statistics

Continues data were summarized by descriptive statistics and comparisons of continuous dependent variables were analyzed using the nonparametric Wilcoxon signed ranks test. All statistical analyses were done with IBM^®^ SPSS^®^ Statistics 24.

## Results

Twenty-seven male patients with a mean age of 55.7 (26–75) years were admitted in the trial. Eight patients had bilateral and nine-teen unilateral hernias, resulting in a total of thirty-five hernias. Body Mass Index mean was 26.3 kg/m^2^ (20–33). No patient was female and no patients had sliding hernias. Other Intra-operative data is shown in Table 1. More extensive information about early complications and 1-year follow-up data has been reported already [17].

**Table 1:** Perioperative data (*n* = 35)

Operating time (min)	54.9 (33–100)^a^
Site of hernia	
Left	18 (51.4)
Right	17 (48.6)
Unilateral	19 (54.3)
Bilateral	16 (45.7)
Size of hernia defect	
< 1.5 cm	5 (14.3)
1.5–3 cm	28 (80.0)
> 3 cm	2 (5.7)

Values in parenthesis are percentages unless indicated otherwise

^a^Values are means (min–max)

### Pain

**Fig. 2:**
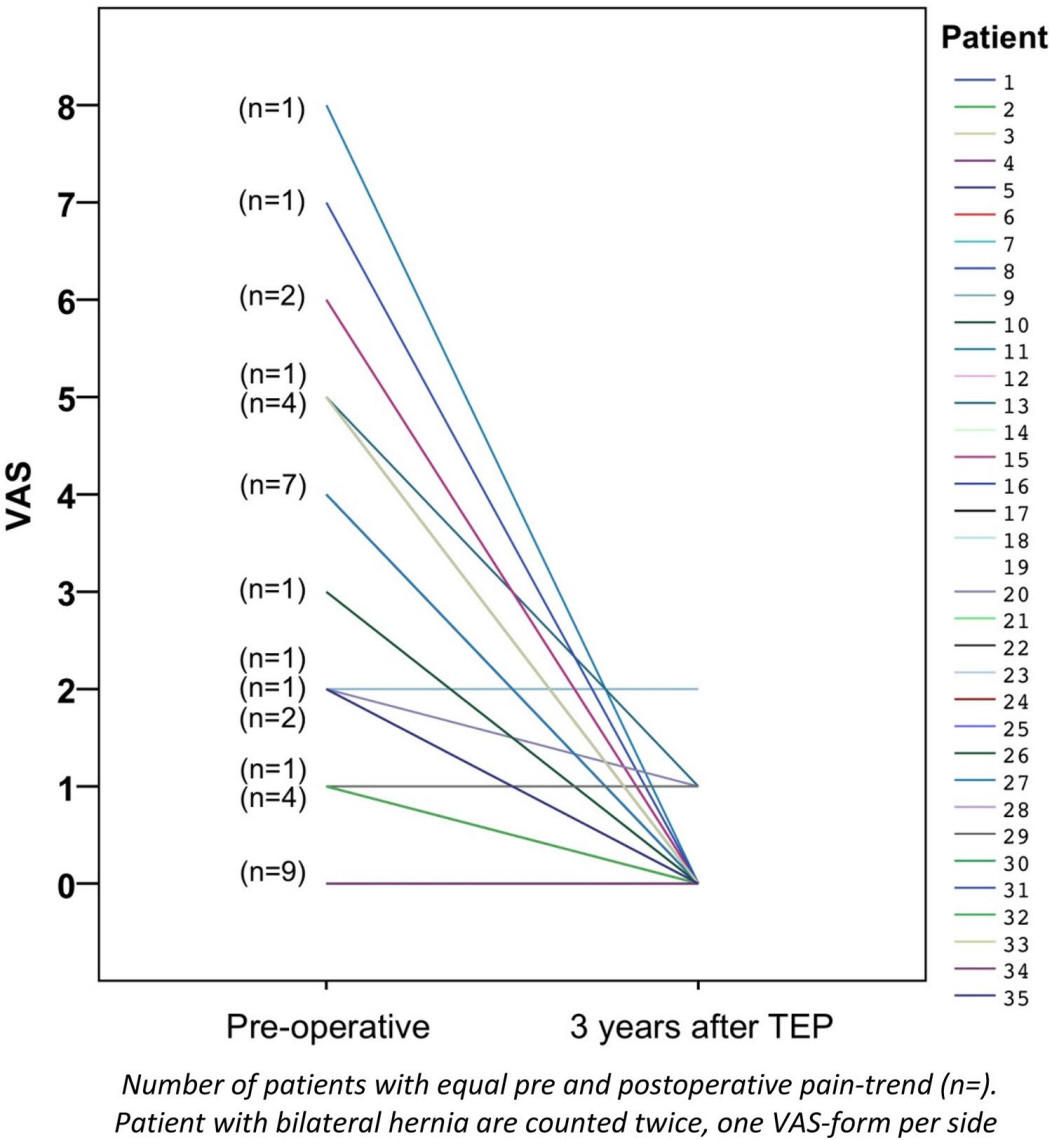
Scatter-plot of the pain on the VAS-scale for each patient before surgery and 3 years after TEP

**Fig. 3:**
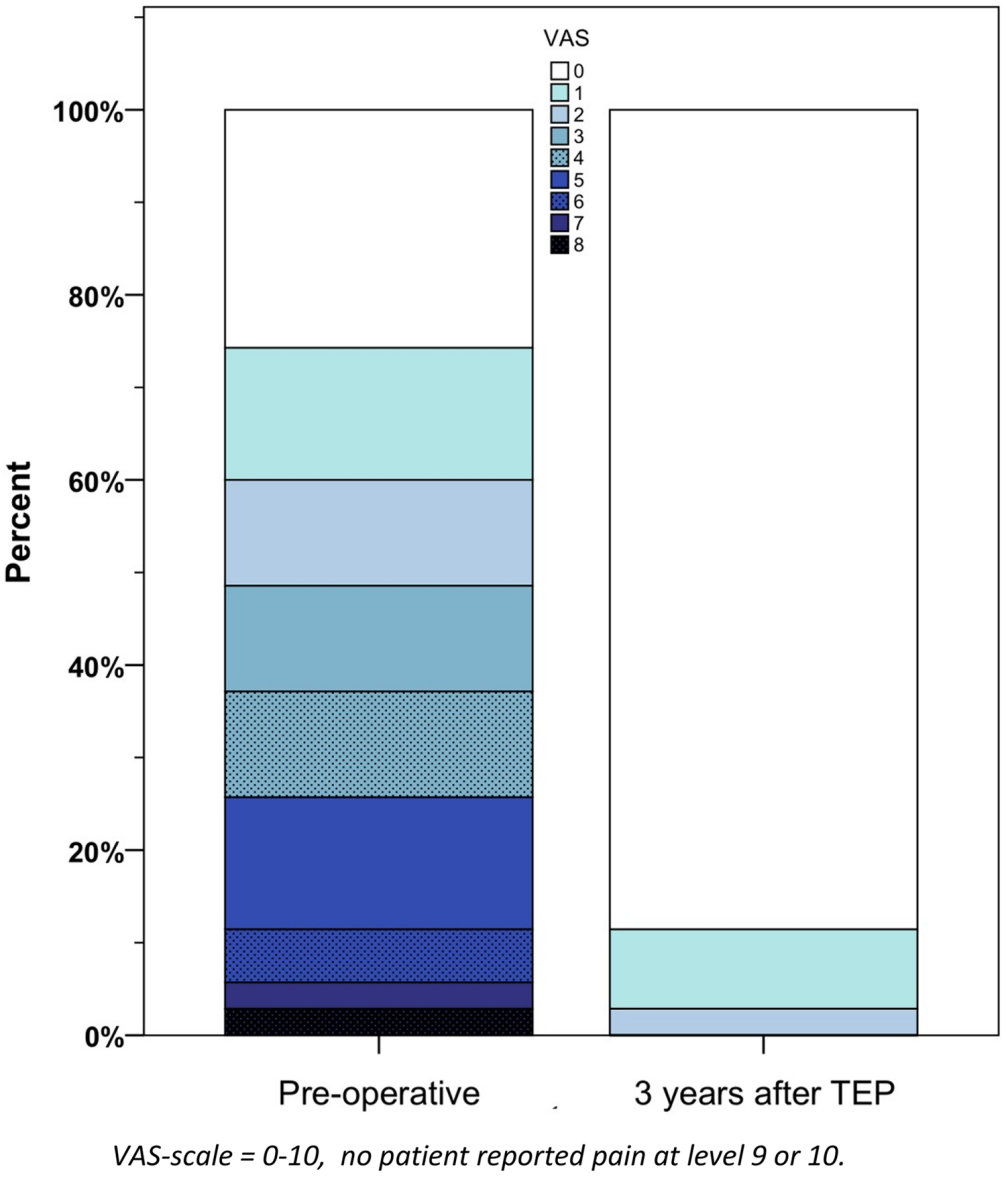
Proportion of patients at different VAS-scale levels before and after surgery

All patients completed the pain questionnaires but one patient did not attend the planned clinical examination for the 3-year follow-up. No patient had chronic post-operative pain as described in the methods at the 3-year follow-up. Almost all the patients had lower VAS score in any activity 3 years following surgery in comparison to the preoperative period (Fig. 2). The mean maximal pain intensity at any activity of the patients assessed by VAS decreased significantly over time from 2.66 pre-operatively to 0.25 3 years postoperatively (Table 2). The proportion of patients who experienced moderate or severe pain (VAS ≥ 3) before the surgery was 48.5%, and then dropped to 0.0% 3 years after the hernioplasty (Fig. 3). No one reported pain at rest and the maximal reported pain on VAS-scale in any activity was 2 at the 3-year follow-up. Patients that reported pain that cannot be ignored or interferes with daily activities in the IPQ questionnaire fell from 24 (69%) preoperatively to 0 (0%) at the 3-year follow-up (Table 3).

**Table 2:** Main of maximal pain at any activity at VAS-scale before and 3 years after surgery

	*N*	Mean	SD	Minimum	Maximum	Median
Preoperatively	35	2.66	2.33	0	8	2.00
3-Year follow-up	35	0.14	0.43	0	2	0.00

Statistical significance of preoperative and postoperative pain using Wilcoxon signed rank test < 0.001

**Table 3:** Number of patients per pain level to the question worst pain past week in the IPQ before and after surgery

Pain estimation	Before surgery	3 Years postoperatively
	*n* = 35	*n* = 35
No pain	10 (28.6)	32 (91.4)
Can easily be ignored	1 (2.9)	3 (8.6)
Cannot be ignored, no interferences with daily activities	9 (25.7)	0 (0.0)
Interferences with daily activities	14 (40.0)	0 (0.0)
Need bed rest because of pain	1 (2.9)	0 (0.0)

Values: number of patients (%). Patients with bilateral hernias are counted twice, a pain questionnaire per side. *IPQ*, Inguinal Pain Questionnaire

### Recurrence

Three patients (8.8%) suffered symptomatic recurrence before the 3-year follow-up. Two patients have been re-operated, one patient for a MIH and the other one for a LIH, 28 and 18 months after primary surgery, respectively. The third patient with recurrence 18 months after primary hernioplasty is not interested in surgery because of absence of bothersome symptoms. At 36-months follow-up no other patients had symptomatic or clinically verified hernias but four new hernia recurrences (11.7%) were found solely on ultrasound.

## Discussion

In this study, only a slowly resorbable implant, without any sutures or tacks, was used to cover the incompetent internal ring of the inguinal canal. At 3-years follow-up the majority of the patients had a competent inguinal canal, without signs of recurrence even using ultrasound examination. Three years after the surgery the mesh should have been fully absorbed [14, 21], therefore the reasonable explanation for the competent inguinal canal is that a new connective tissue stimulated by the mesh has patched the hernia defect [21].

Some biological meshes used in hernia surgery have shown likewise acceptable results on recurrences [22–24]. Those prostheses are enzymatically degraded after implantation [25, 26] which reinforces the thesis that connective tissue defects may sometimes only need temporary restoration, not permanent repair.

### Recurrences and degradable meshes

The idea of using a synthetic slowly degradable mesh in hernia surgery is based on the hypothesis that the body just needs a matrix to stimulate the formation of a fibrous sheet to cover the hernia defect and reinforce the abdominal wall. This concept presupposes a normal function of the regenerative connective tissue including collagen [26, 27]. Therefore patients with collagen defects could run a larger risk of recurrence if they were to have an Inguinal Hernia repair with resorbable meshes. An abnormal connective tissue remodeling could also explain why patients with MIH, which is associated with collagen failure [28–31], have higher recurrence rate compared to patients with LIH after hernioplasty [2, 3].

All the patients with palpable recurrences in this study had the first symptoms of recurrences at least 18 months after surgery. At this time the mesh should have lost the mechanical stability to contain the abdominal pressure [21]. If an incorrect placement of the mesh during the surgery can be excluded, a possible reason could be that those patients had a deficient rebuilding of the scar tissue around the implant. An evaluation of the collagen status of the patients selected for this trial was not planned; thus, it is not possible to confirm that patients with recurrences had altered collagen remodeling compared with patients without recurrences. A difficulty in the evaluation of the collagen status lies in that there are no well-established biomarkers in blood samples to know if a patient has collagen or connective tissue alterations or not [32, 33].

One of the patients with recurrence was operated primary for bilateral hernias. The patient had a MIH recurrence solely in the side with the primary bigger defect but no recurrence has been found yet on the other side. If this situation does not change in the future, it would mean that not only connective tissue deficiencies are implicated in the recurrences, but other factors, like the size of the hernia defect, can play a role for hernia recurrences.

This study found an incongruence between ultrasound verified recurrences and recurrences found by palpation at 3-year follow-up. Patients with recurrences solely confirmed by ultrasound did not have any symptoms. The reliability of ultrasound in the diagnosis of symptomatic but no palpable hernias is well known [34]; however, the sensitivity and the specificity of ultrasound in the diagnosis of hernia recurrence in asymptomatic and non-palpable hernias after hernioplasty have not been investigated [35–37]. Additionally, some trials have shown that occult hernias found during laparoscopy have not had any clinical relevance [38]. For this reason it is difficult to conclude that the hernia recurrences solely verified by ultrasound in this study, have a clinical value for the patients. A longer follow-up is required for those patients in order to know the clinical value of a recurrence confirmed solely by ultrasound. A longer follow up is necessary even for the rest of patients because is well-known that recurrence occur even decades after surgery [3, 39].

### Chronic post-operative pain and degradable meshes

There is an enormous variation of the rate of pain after hernioplasty in the literature (20–45%) [40, 41]. This variation could be explained by the fact that many aspects are not uniform in those pain studies: method and timetabling for pain measurement, definition of chronic pain and its distinction from discomfort, selection criteria of the patients, assessment or not of the pre-operative pain among others [40]. All this impedes a comparison of the risk of CPP with similar trials.

In the current study, we have excluded patients with preexisting inguinal pain conditions unrelated to the hernia. However, 68.6% of the included patients suffered from clinically significant inguinal pain preoperatively, which is considered a risk factor for CPP [5, 41]. Despite this risk factor for CPP, the present trial found a very low risk (0%) of CPP even for patients with pre-operative inguinal pain. Several factors in combination can possibly explain those good results on pain. However, there are two aspects that may play a greater role. On one hand, TEP per se has a lower risk of CPP than Lichtenstein according to several studies [15, 16, 42, 43]. On the other hand, resorbable meshes do not produce chronic foreign body inflammation once the mesh has been degraded [21, 26].

### Limitations, strengths and implications of the study

This was the first study using a synthetic slowly resorbable mesh in the TEP procedure in human, thus it was planned as a safety and feasibility study. Consequently the limitations of the study lie in the non-randomization and in the small number of patients. However, the assessment of pre-operative and post-operative pain using diverse validated pain questionnaires, the rigorous assessment of possible recurrence with ultrasound and physical examination and the long follow-up strengthens the results of the present study. The role of slowly resorbable meshes on CPP in LIH repair cannot be elucidated from this study but the results regarding chronic post-operative pain in these patients are so satisfactory that it could motivate larger, randomized studies in a more selected patient population in order to confirm these findings. Given that one part of the population with inguinal hernias have collagen deficiencies [28, 30, 31, 33], the risk of recurrences could increase if absorbable meshes are used in the hernia repair of those patients. On the other hand patients with inguinal hernias without collagen deficiencies could be more suitable for a randomized study using synthetic long-term degradable meshes in the hernia repair.

## Conclusion

TEP repair in patients with LIH using a synthetic long-term resorbable mesh was found to be encouraging respecting chronic post-operative pain at 3-year follow up but at the cost of an increased risk of recurrence. Longer follow-up is necessary to establish the risk of recurrence at an even longer follow-up time than 3 years. Randomized control studies with standard mesh are necessary to know the real effect of slowly degradable implants on CPP. But such studies need more selective inclusion criteria of the patients in order to increase the potential benefits on CPP without raising the risk of recurrence in those patients.
